# Study on Purification, Identification and Antioxidant of Flavonoids Extracted from *Perilla leaves*

**DOI:** 10.3390/molecules28217273

**Published:** 2023-10-26

**Authors:** Hui Li, Jiayu Lin, Baoqing Bai, Tao Bo, Yufei He, Shanhong Fan, Jinhua Zhang

**Affiliations:** 1 College of Life Sciences, Shanxi University, Taiyuan 030006, China; lh_0905@163.com (H.L.); ljy141024520@163.com (J.L.); baoqingbai@sxu.edu.cn (B.B.); 2 Shanxi Key Laboratory of Research and Utilization of Characteristic Plant Resources, Shanxi University, Taiyuan 030006, China; 3 Institute of Biotechnology, Shanxi University, Taiyuan 030006, China; botao@sxu.edu.cn; 4 Shanxi Food Research Institute Co., Ltd., Taiyuan 030024, China; sxfood@126.com

**Keywords:** *Perilla leaves*, flavonoids, isolation and purification, activity study

## Abstract

The flavonoids from *Perilla leaves* were extracted using flash extraction assisted by ultrasonic extraction with ethanol. Subsequently, macroporous resin was employed for the isolation and purification of these flavonoids, followed by an investigation into their antioxidant activity. The process conditions for the extraction of flavonoids from *Perilla leaves* were designed and optimized using a one-way experiment combined with a response surface methodology. The optimal extraction conditions were determined as follows: the liquid–solid ratio was 20:1, ethanol volume fraction of 60%, ultrasound temperature of 60 °C, ultrasound time of 10 min and flash evaporation time of 60 s. The optimal extraction rate of flavonoids is 9.8 mg/g. In terms of separation and purification, a high-performance macroporous resin (HPD450 resin) with high purification efficiency was selected through static analysis and adsorption experiments. The optimal enrichment conditions were as follows: loading concentration of 0.06 mg/mL, optimal loading concentration of 20 mL, elution concentration of 70% and 76 mL, providing a reference for the further development and utilization of *Perilla leaf* flavonoids.

## 1. Introduction

*Perilla* (L.) Britt is an annual herbaceous plant belonging to the Labiatae family of the genus *Perilla*, also known as red *Perilla leaves* or suzi [1,2]. *Perilla*, one of the initial 60 medicinal and edible plants officially recognized by the Ministry of Health, holds a significant position as a traditional versatile economic plant in China [3].

Several current studies have reported that *Perilla* contains a variety of bioactive components such as volatile oils, flavonoids, phenolic acids, terpenoids, anthocyanins and polysaccharide glycoside compounds [4], its biological activities mainly include antioxidant, anti-inflammatory, bacteriostatic, anti-depression, anti-tumor, sedative effects and so on [5]. Zheng Yunfeng et al. [6] identified seven flavonoids, including baicalein-7-O-diglucuronide, lignan-7-O-diglucuronide, apigenin-7-O-diglucuronide, baicalein-7-O-glucuronide, lignan-7-O-glucuronide, apigenin-7-O-glucuronide and lignan, by comparing the differences in the biologically active constituents of the two *Perilla leaf* compounds. This study also elucidated that the differences in antioxidant activity of different varieties of *Perilla leaves* mainly depended on the changes in flavonoid content. Tao Jiang et al. [7] reported that the main components of flavonoids in *Perilla leaves* were chrysoeriol, apigenin, hesperetin, quercetin, kaempferol, etc., and elucidated the existence of variability in the content of flavonoids in the *leaves* of *Perilla* of different varieties.

Flavonoids, widely present in nature in plants, are secondary metabolites in plants, and are therefore also known as plant flavonoids [8]. This class of compounds not only has a large number, but also has a complex structure and many important physiological activities [9]. In other words, flavonoids have multiple functions such as antioxidant, anti-inflammatory, anticancer, hypotensive, hypolipidemic and antibacterial [10]. Ayaka Nakajima et al. [11] purified flavonoid constituents such as 5,8-dihydroxy-7-methoxyflavanone, negletein (5,6-dihydroxy-7-methoxyflavone), luteolin, apigenin, esculetin and protocatechuic acid from the ethyl acetate-soluble fraction of *Perilla leaves*, and the results suggest that these constituents may be involved in the anti-inflammatory effects of *Perilla leaves*. Kaewseejan et al. [12] showed that the content and composition of flavonoids in plants may play an important role in antioxidant activity.

This study used a flash extraction-assisted ethanol ultrasound method to extract total flavonoids from Shanxi *Perilla leaves*. The single factor experiment clarified the values of the factor conditions affecting the extraction rate of flavonoids, and the process conditions for the extraction of total flavonoids from *Perilla leaves* were optimized using a response surface methodology, and the optimum extraction conditions were determined. On this basis, the flavonoids in *Perilla leaves* were isolated and purified using macroporous resins. Finally, the antioxidant capacity of purified total flavonoids from *Perilla leaves* was evaluated through in vitro antioxidant tests, which promoted the comprehensive development of *Perilla leaves* and expanded their application potential.

## 2. Results and Analysis

### 2.1. Optimization of Extraction Process of Flavonoids from Perilla leaves

#### 2.1.1. Single Factor Test Results

##### Effect of Liquid–Solid Ratio on Extraction Rate of Flavonoids from *Perilla leaves*

**Figure 1:**
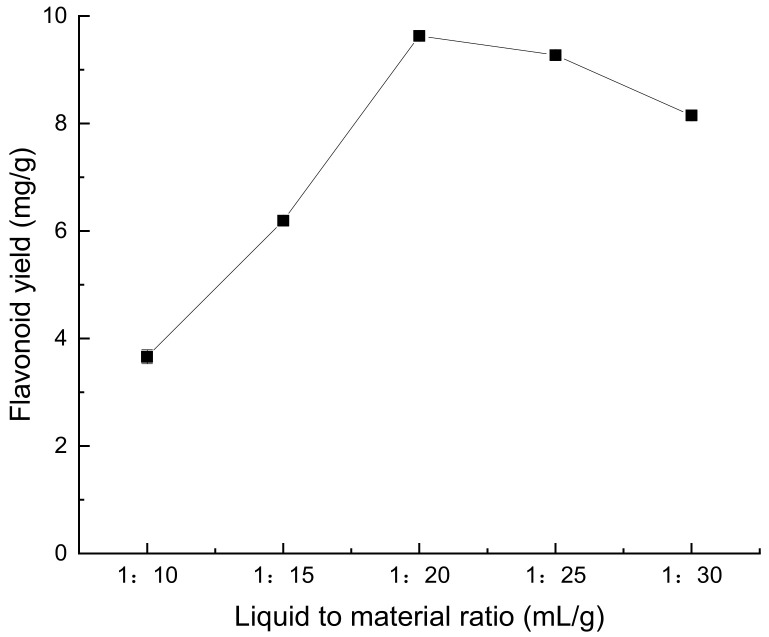
Effect of liquid–solid ratio on extraction rate of flavonoids from *Perilla leaves*.

The highest extraction rate of flavonoids from *Perilla leaves* was observed when the liquid–solid ratio was 20:1 (mL/g), as depicted in Figure 1. When the liquid-to-material ratio exceeds 20:1, there is a reduction in the yield of flavonoids. The reason may be that the right amount of solvent is conducive to the dissolution of flavonoids, while excessive solvent will have adverse effects on the mass and heat transfer of the extraction system, and then lead to the decrease of the yield of flavonoids [13].

##### Effect of Ethanol Volume Fraction on Extraction Yield of Total Flavonoids from *Perilla leaves*

**Figure 2:**
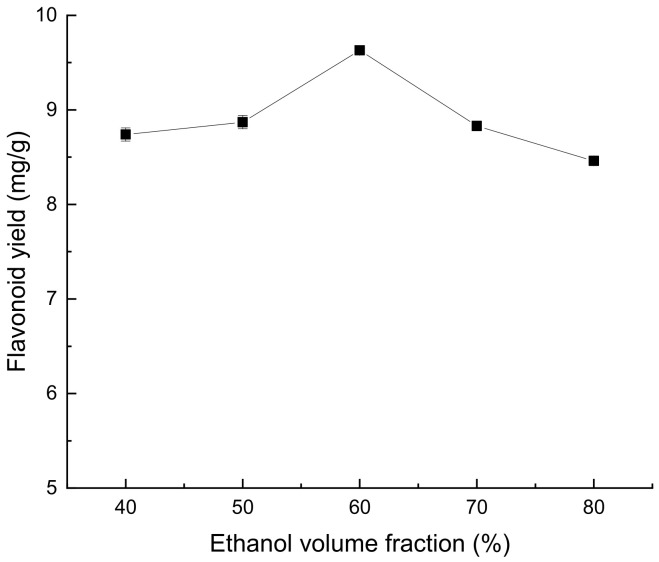
Effect of ethanol volume fraction on extraction yield of total flavonoids from *Perilla leaves*.

According to Figure 2, the extraction rate of flavonoids from *Perilla leaves* increased when the volume fraction of ethanol ranged between 40% and 60%. However, when the volume fraction of ethanol exceeded 60%, a higher volume fraction would lead to a decrease in the extraction yield. This may be because, when the ethanol volume fraction is low, the solubility of flavonoids in *Perilla leaves* will increase with the increase of the ethanol volume fraction, resulting in an increase in the extraction rate. When the volume fraction of ethanol is too large, the leaching amount of some alcohol-soluble impurities and pigments will also increase. These compete with flavonoids and bind to ethanol–water molecules, resulting in reduced extraction rates of flavonoids [14].

##### Effect of Ultrasonic Temperature on Extraction of Flavonoids from *Perilla leaves*

**Figure 3:**
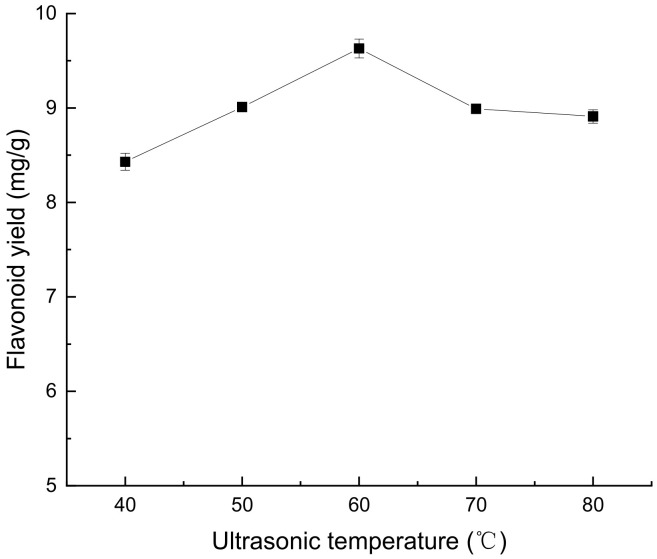
Effect of ultrasonic temperature on extraction of flavonoids from *Perilla leaves*.

According to Figure 3, an increase in ultrasound temperature will increase the diffusion rate of solvents and the solubility of *Perilla leaf* flavonoids, which will lead to an increase in the extraction rate. Therefore, the peak yield of *Perilla leaf* flavonoids was observed at an ultrasonic temperature of 60 °C. This may be because more flavonoids are released from *Perilla leaves* at the optimal temperature. Further heating may lead to the destruction of the structure of the flavonoids [15], so a further increase in temperature will reduce the flavonoid extraction rate.

##### Effect of Ultrasonic Time on Extraction of Flavonoids from *Perilla leaves*

**Figure 4:**
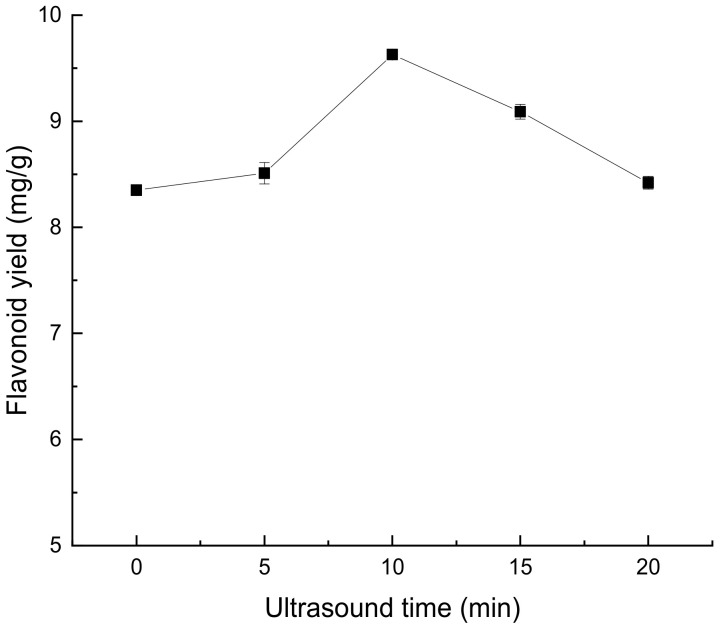
Effect of ultrasonic time on extraction of flavonoids from *Perilla leaves*.

The length of ultrasonic treatment time had a certain effect on the yield of total flavonoids in *Perilla leaves*, as shown in Figure 4. From 0 to 10 min, with the increase of time, the extraction rate of flavonoids from *Perilla leaves* also increased. At 10 min, the extraction rate of flavonoids from *Perilla leaves* reached the maximum, which may be due to the longer contact time between solid and extraction solvent, which promoted the diffusion of flavonoids. However, beyond 15–20 min, the extraction rate decreases, which may be due to the extended ultrasonic extraction time, which produces additional mechanical effects on the flavonoids, which in turn leads to the degradation of the flavonoids [16].

##### Effect of Flash Time on Extraction of Flavonoids from *Perilla leaves*

**Figure 5:**
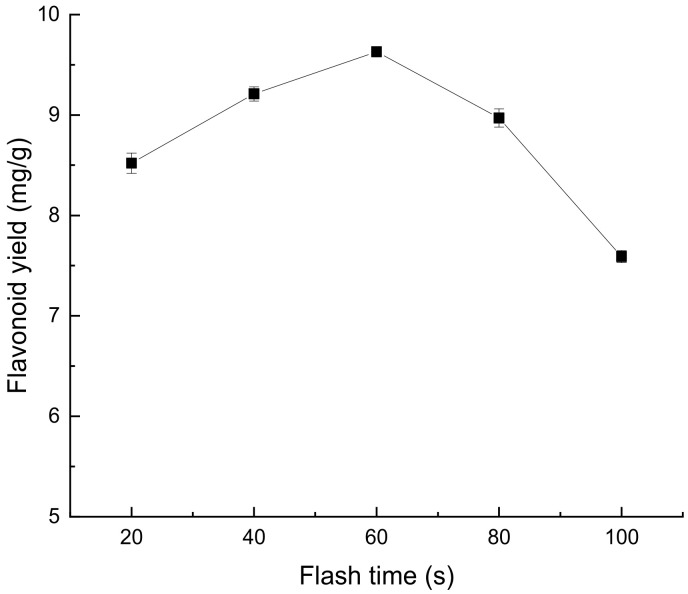
Effect of flash time on extraction of flavonoids from *Perilla leaves*.

As can be seen from Figure 5, during the period of 20~60 s, the extraction rate of flavonoids in *Perilla leaves* showed a positive relationship with the flash extraction time, and the extraction rate reached the maximum value in 60 s. Within 60–100 s, the yield of flavonoids in *Perilla leaves* decreased with time. This may be because the extraction time is too long, the extraction temperature rises and this accelerates the oxidation of flavonoids in *Perilla leaves* or causes the change of chemical composition, thus reducing the extraction rate of flavonoids in *Perilla leaves* [17,18].

#### 2.1.2. Box-Behnken Test Results

Based on the experimental data (Table 1) on the extraction rate of flavonoids from *Perilla leaves*, a quadratic multiple regression equation was established by analyzing the data using the response surface software Design-Expert V 8.0.6:

Y flavonoids extraction rate (%) = 0.038 + 9.79 + 1.27 × A − 0.038 × B + 0.070 × C + 0.14 × A × B + 0.040 × A × C + 0.055 × B × C − 1.48 × A^2^ − 0.37 × B^2^ − 0.35 × C^2^ (1)

Table 2 shows the models (*p* < 0.0001), indicating that the model had a significant effect on the extraction rate of flavonoids from *Perilla leaves*, where the missing fitting term (*p* = 0.0612 > 0.05) showed that the fitting equation could be used to predict the optimal extraction process of flavonoids from *Perilla leaves*. The coefficient of determination, R^2^, of the equation is 0.9995 > 0.9, the corrected regression coefficient RAdj2 is 0.9988, which further proves that the regression equation can effectively explain 99.88% of the variation of flavonoid extraction rate [19]. In addition, in the first-order term of the regression equation, the liquid–solid ratio (A), ethanol volume fraction (B) and ultrasound temperature (C) significantly affect the yield of flavonoids in *Perilla leaves* (*p* < 0.05). In the interaction term, the interaction between the liquid–solid ratio and the ethanol volume fraction (AB), as well as the interaction between the ethanol volume fraction and the ultrasound temperature (BC), are important factors affecting the total flavonoid yield of *Perilla leaves*. The quadratic terms (A2, B2, and C2) all have extremely significant effects (*p* < 0.01); according to the F and *p* values [20], the degree of impact on the total flavonoid yield in *Perilla leaves* can be determined as follows: liquid–solid ratio (A) > ultrasonic temperature (C) > ethanol volume fraction (B).

The Design-Expert software optimized the process conditions for extracting flavonoids from *Perilla*. The optimal extraction conditions are: liquid–solid ratio 20:1 (mL/g), ethanol volume fraction 60%, ultrasound temperature 60 °C and maximum theoretical value of total flavonoid extraction rate 9.82 mg/g. The optimal conditions were applied in the actual experiment, and the average yield of flavonoids that could be extracted was 9.8 mg/g, close to the theoretical value, which verified the accuracy of the model and the reliability of the process scheme. Therefore, the model could be used to extract flavonoids from *Perilla leaves*.

### 2.2. Separation and Purification of Flavonoids from Perilla leaves

#### 2.2.1. Static Adsorption and Resolution of Resin

**Figure 6:**
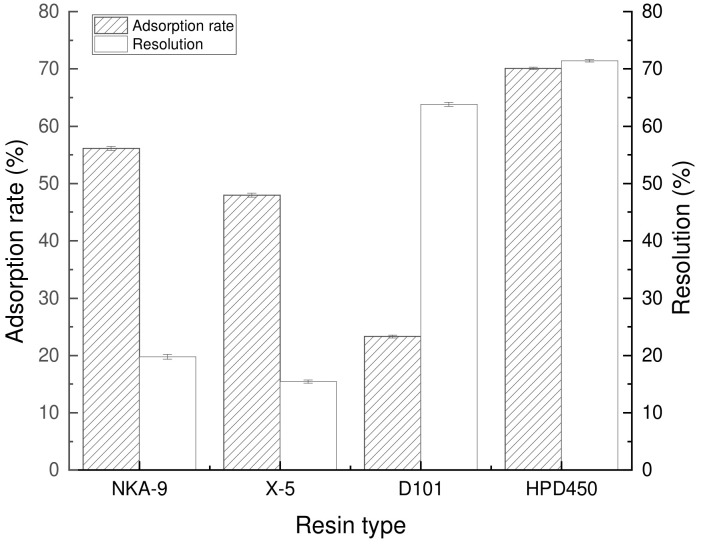
The adsorption and resolution capacity of four resins for flavonoids.

Figure 6 shows the adsorption and resolution diagrams of four macroporous resins, namely NKA-9, X-5, D101 and HPD450. Due to the distinct physical properties of macroporous resins, their adsorption capacity for flavonoids from *Perilla leaves* varies, thereby resulting in divergent adsorption and resolution characteristics. Adsorption rate: HPD450 > NKA-9 > X-5 > D101; the resolution rate was HPD450 > D101 > NKA-9 > X-5.

The adsorption and resolution rates of HPD450 resin were higher than those of the other three resins. The adsorption and resolution abilities of HPD450 resin were not only related to solution concentration, temperature and other factors, but also restricted by physical and chemical properties such as specific surface area and pore size of the macroporous resin. The larger the specific surface area of the resin, the higher the adsorption capacity. A larger specific surface area of HPD450 resin is conducive to the adsorption of flavonoids from *Perilla leaves* [21]. On the other hand, according to the molecular mass size of the compounds to be adsorbed, an appropriate pore size resin should be selected to achieve the purpose of effective separation, according to the law of “the same kind of dissolution of the same kind”, the resin with higher polarity has a better adsorption capacity for high polarity and polar substances [22,23]. And the HPD450 resin pore size is the smallest; it belongs to the medium polar resin. In summary, HPD450 resin was selected as the resin material for subsequent purification work.

#### 2.2.2. Separation and Purification of Flavonoids from *Perilla leaves*

**Figure 7:**
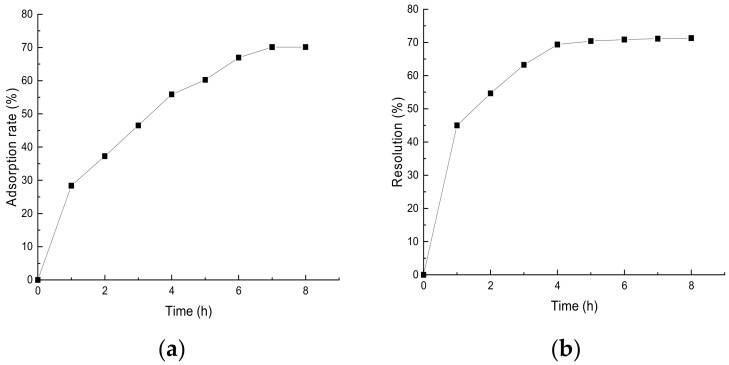
Static adsorption and analytical dynamic curves of HPD450 resin. (**a**) represents the adsorption rate of HPD450 resin; (**b**) represents the resolution of HPD450 resin.

Adsorption kinetics describes the adsorption rate and adsorption time of the target substance on the surface of the adsorbent [24]. The adsorption time and adsorption rate of the macroporous resin were used as evaluation indexes to investigate the adsorption and separation performance of HPD450 resin on *Perilla leaf* flavonoids. As shown in Figure 7a, it can be concluded that the static adsorption of total flavonoids from *Perilla leaves* by HPD450 resin was of medium rate equilibrium type. The resin was in the rapid adsorption stage within 0–6 h at the beginning of adsorption, and in the slow adsorption stage from 6–8 h. After 8 h, the resin basically reached the adsorption equilibrium state. It can be seen from Figure 7b that the resolution rate of HPD450 resin increased rapidly from 0 to 2 h, and slowed down from 2 to 5 h. After 5 h, the resolution reached an equilibrium point. Considering various indicators, HPD450 resin not only has strong adsorption capacity, but also has a high resolution rate and easy elution, Therefore, HPD450 resin is the best type of resin for the separation and purification of flavonoids from *Perilla leaves*.

#### 2.2.3. Optimization of the Enrichment Process of HPD450 Macroporous Resin

##### The Influence of Sample Amount on the Resolution Rate of HPD450 Macroporous Resin

**Figure 8:**
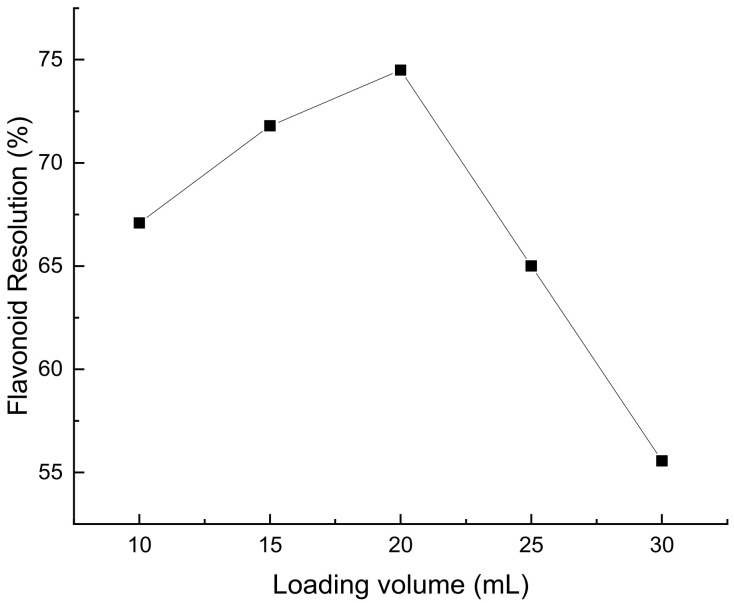
The effect of sample loading on the resolution rate of flavonoids from *Perilla leaves*.

It can be seen from Figure 8 that, when the sample volume was less than 20 mL, the flavonoid resolution rate increased with the increase of the sample volume. A sampling volume less than 20 mL leads to resin waste and low purification efficiency. This may be due to the fact that the maximum amount of adsorbable macroporous resin is much larger than the amount of flavonoids in the sample solution. When the loading volume was 20 mL, the highest flavonoid resolution rate was 74.49%. When the upper sample volume is larger than 20 mL, the resolution of flavonoids gradually decreases. This may be due to the excessive content of flavonoids in the sample, which are no longer adsorbed by the resin column after adsorption saturation, resulting in the loss of sample liquid and a decrease in the recovery rate [25].

##### Influence of Eluent Concentration on Resolution Rate of HPD450 Macroporous Resin

**Figure 9:**
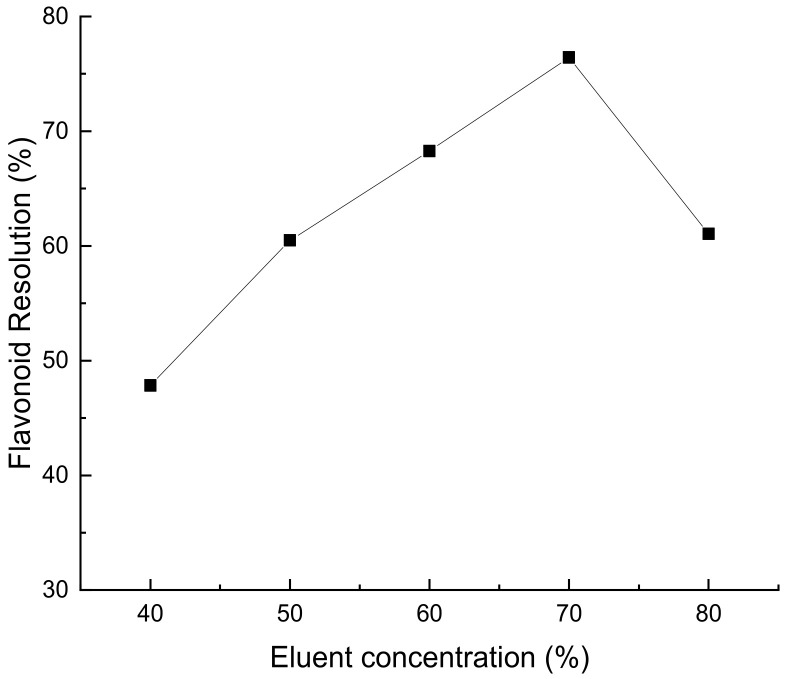
Effect of eluent concentration on resolution rate of *Perilla leaf* flavonoids.

As shown in Figure 9, a 70% ethanol solution has the best elution effect. This may be due to the presence of a certain amount of water in the eluent, which facilitates the elution of flavonoids. The recovery of flavonoids from *Perilla leaves* was low when the volume fraction of ethanol was less than 70%. Two reasons are analyzed, one is due to the fact that flavonoids will be adsorbed on the macroporous resin due to hydrogen bonding with the resin, and the other is that the polarity of the eluent is less than the force between the macroporous resin and the sample solution [26]. The final result is that flavonoids are not easily absorbed and eluted by low volume fraction ethanol solutions.

##### Effect of Eluate Volume on the Concentration of Flavonoids in *Perilla leaves*

**Figure 10:**
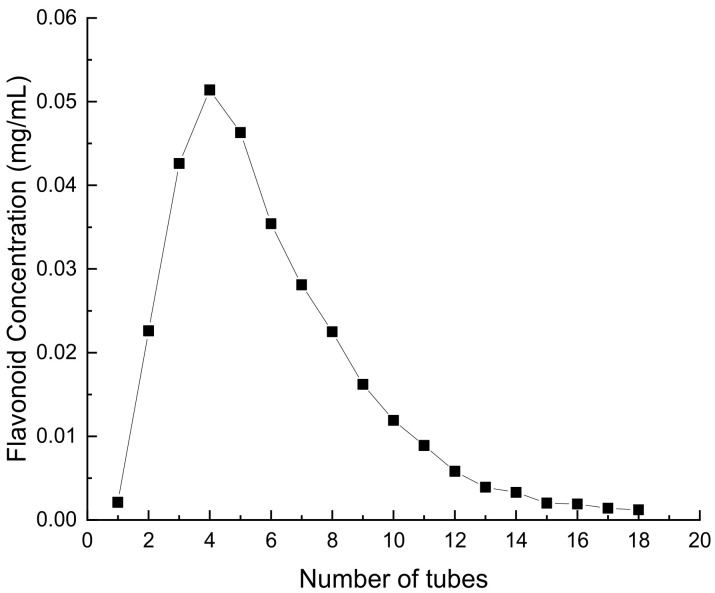
Effect of eluent volume on flavonoid concentration in *Perilla leaves*.

The concentration of flavonoids exhibited a parabolic trend, characterized by an initial increase followed by a subsequent decrease, as depicted in Figure 10. The collection of flavonoids from *Perilla leaves* was concentrated in 2–16 tubes; 2–4 of the tubes were 2 mL, and the rest of the tubes were 5 mL. When the number of tubes reached 18, basically no flavonoids could be detected in the eluent, indicating that all the flavonoids from *Perilla leaves* had been eluted. Therefore, the optimal volume of eluate was 76 mL [27].

### 2.3. Identification of Flavonoid Compounds in Perilla leaves

**Figure 11:**
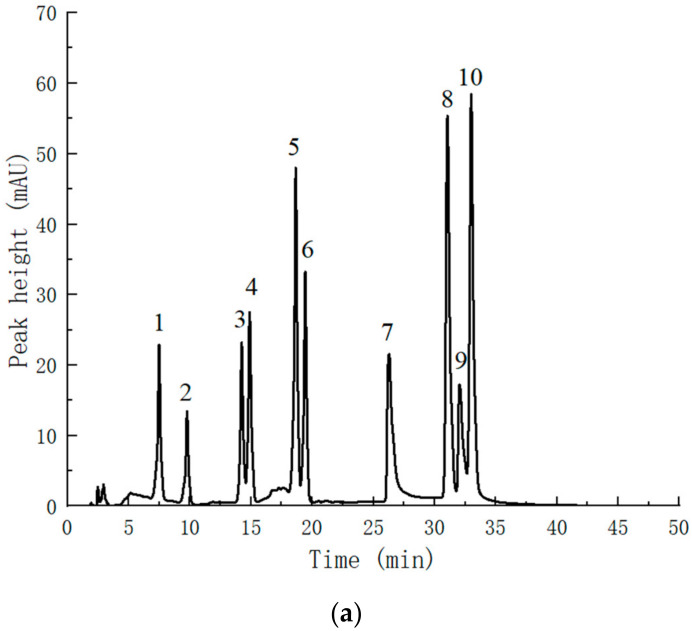

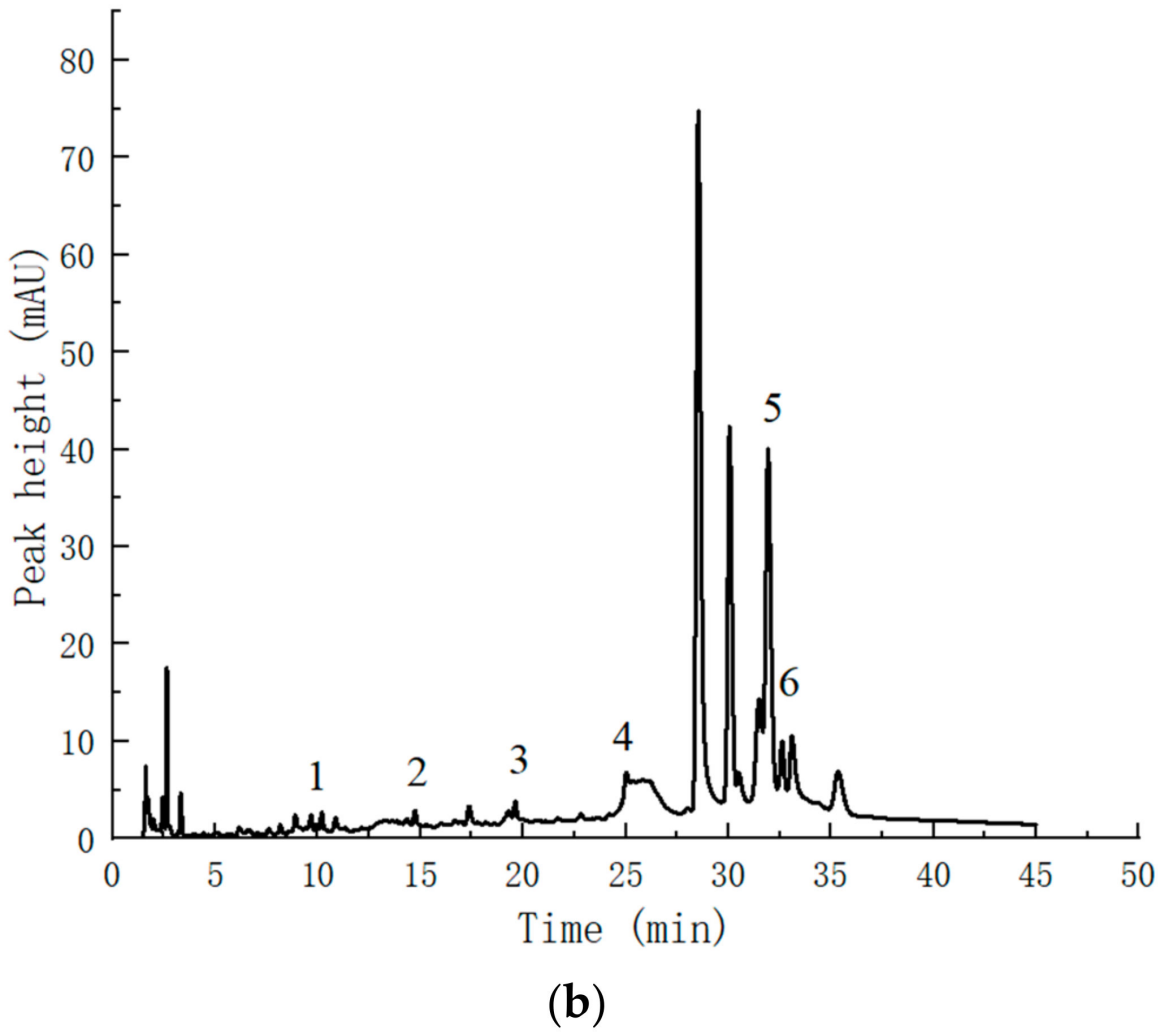
(**a**) Liquid chromatogram of the standard. 1. catechin; 2. epicatechin; 3. rutin; 4. hypericin; 5. naringin; 6. hesperidin; 7. luteolin; 8. naringenin; 9. kaempferol; 10. hesperetin. (**b**) Liquid chromatogram of *Perilla* leaf flavonoids. 1. epicatechin; 2. hypericin; 3. hesperidin; 4. luteolin; 5. kaempferol; 6. hesperetin.

According to the liquid chromatography separation conditions provided in Section 3.2.4, the analysis results are shown in Figure 11b. Compared with the standard chromatogram (Figure 11a), *Perilla leaves* contain flavonoids such as epicatechin, hypericin, hesperidin, kaempferol and hesperetin. The flavonoid constituents identified in the present study have been shown to possess some degree of antioxidant and antiviral activity by Panche, A. et al. [28]. According to the single-standard liquid chromatogram, it is speculated that the *Perilla leaf* flavonoids also contain flavonoids such as quercetin and apigenin. Qualitative analysis using high performance liquid chromatography alone has certain limitations, and it is difficult to obtain all the information of flavonoids. Therefore, it is necessary to use other analytical methods such as mass spectrometry and nuclear magnetic resonance for more accurate compound identification.

### 2.4. Study on Antioxidant Activity of Flavonoids from Perilla leaves

#### 2.4.1. DPPH Free Radical Scavenging Activity of *Perilla leaf* Flavonoids

**Figure 12:**
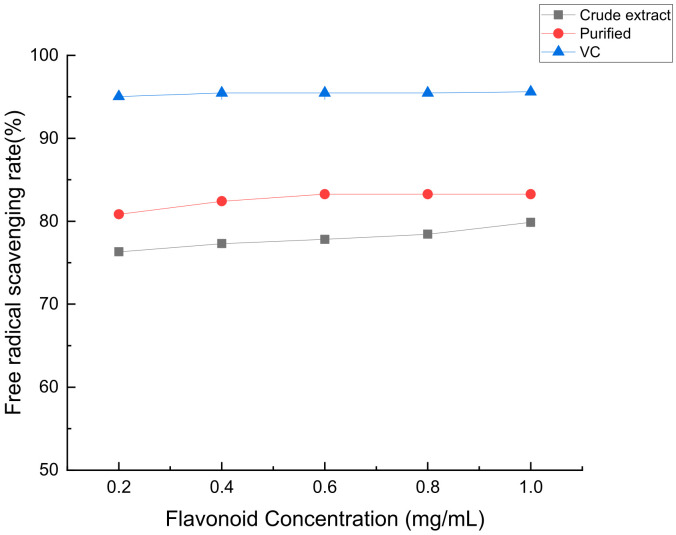
The scavenging ability of crude flavonoids, refined flavonoids and VC from *Perilla leaves* to DPPH free radicals and hydroxyl radicals.

According to Figure 12, as the concentration of flavonoids in *Perilla leaves* increases, its scavenging rate of the DPPH free radical gradually increases, its antioxidant capacity size: coarse extract < purification < VC.

#### 2.4.2. Hydroxyl Radical Scavenging Ability of *Perilla leaf* Flavonoids

**Figure 13:**
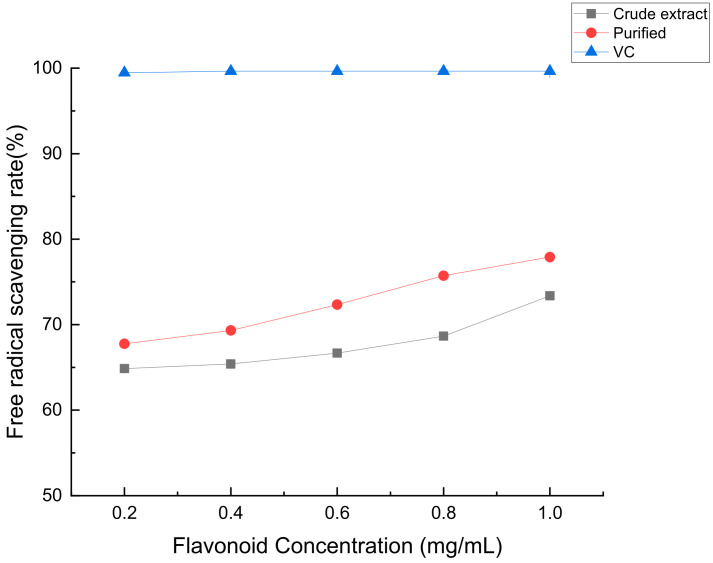
The scavenging ability of crude flavonoids, refined flavonoids and VC from *Perilla leaves* to hydroxyl radicals.

According to Figure 13, as the concentration of flavonoids in *Perilla leaves* increases, its scavenging rate of hydroxyl radicals gradually increases, and then its antioxidant capacity is: crude extract < purified product < VC.

#### 2.4.3. ABTS Free Radical Scavenging Ability of *Perilla leaf* Flavonoids

**Figure 14:**
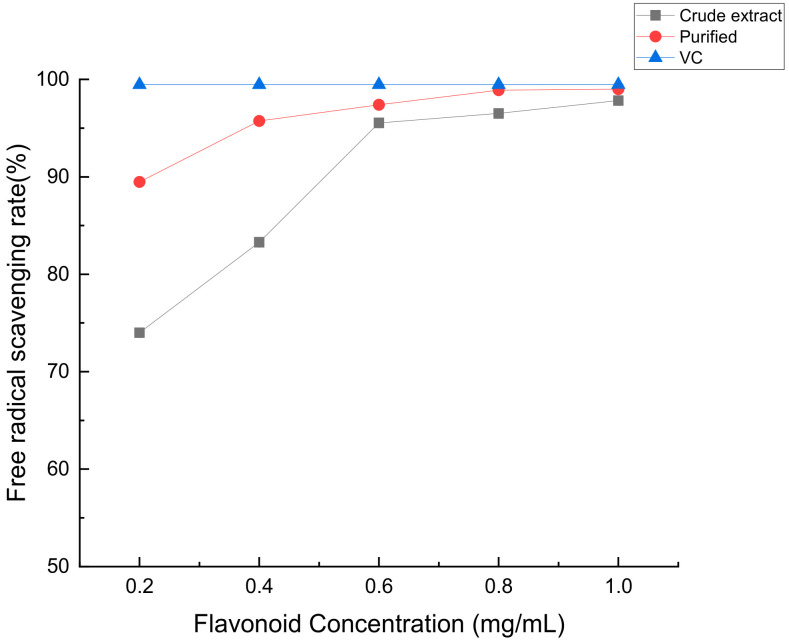
The scavenging ability of crude flavonoids, refined flavonoids and VC from *Perilla leaves* to ABTS free radical.

In the presence of oxidants, ABTS will oxidize to ABTS+• free radicals, and the solution will appear green. When antioxidants are added to the system, the amount of ABTS+• production decreases, and the solution color will become light, from dark green to light green or even light yellow gradually [29]. As can be seen from Figure 14, the ABTS scavenging rate increased with the increase of flavonoid concentration, and the order of scavenging ability was VC > purified > crude extract. When the flavonoid concentration reached 1.0 mg/mL, both the crude extract and purified substance demonstrated ABTS free radical scavenging abilities comparable to that of the positive control VC.

#### 2.4.4. Effect of *Perilla leaf* Flavonoids on Fe^3+^ Reducing Power

**Figure 15:**
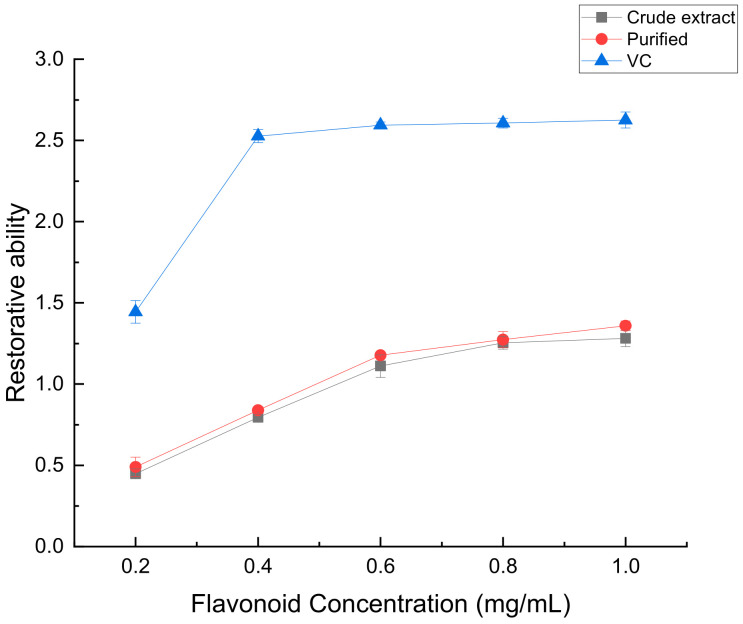
Iron reduction ability of crude flavonoids, refined flavonoids and VC from *Perilla leaves*.

In this experiment, the sample can react with red blood salt to produce yellow blood salt, and then react with Fe^3+^ to produce Prussian blue. The production amount of Prussian blue reflects the reducing power. The greater the absorbance value, the more pronounced the reducing power exhibited by the sample [30]. From Figure 15, it is evident that the reduction ability of VC outperformed both the crude extract and purified forms. However, the purified form exhibited a slightly higher reduction ability compared to the crude extract. This may be because some antioxidant substances were lost in the purification process, resulting in little difference in the reduction ability of flavonoids contained in crude extract and purified.

## 3. Materials and Methods

### 3.1. Materials and Instruments

Materials and reagents: *Perilla leaves*, varieties are one-sided red, from Jincheng, Shanxi Province, harvested in September, washed, dried, crushed, after 80 mesh sieved for use; rutin (>95%), absolute ethanol, sodium nitrite, aluminum nitrate, sodium hydroxide, DPPH, ABTS, ferric chloride, salicylic acid, trichloroacetic acid, potassium ferricyanide, ferrous sulfate hydrogen peroxide were all analytically pure.

Instruments and equipment: electronic balance, traditional Chinese medicine pulverizer, low-speed centrifuge, experimental flash extractor, ultrasonic cleaner, UV spectrophotometer, vacuum freeze dryer, rotary evaporation instrument, constant temperature water bath.

### 3.2. Experimental Methods

#### 3.2.1. Extraction Technology of Flavonoids from *Perilla leaves*

The method for extracting flavonoids from *Perilla leaves* was referenced from Yu, J et al. [31] with minor adjustments. The mass concentration of flavonoids calculated by rutin is the horizontal coordinate, absorbance is the vertical coordinate, and the standard curve is drawn.

The standard curve of rutin was y = 12.129x − 0.0064, R^2^ = 0.9995, the extraction efficiency formula for obtaining flavonoids from *Perilla leaves* was:

W = CVn/M(2)

where W is the extraction efficiency of flavonoids from *Perilla leaves* (mg/g); C represents the concentration of flavonoids in the sample, measured in milligrams per milliliter (mg/mL); V denotes the overall volume of the sample solution, expressed in milliliters (mL); M refers to the weight of *Perilla leaf* powder, specified in grams (g).

#### 3.2.2. Optimization of Extraction Process of Flavonoids from *Perilla leaves*

Accurately measure 1.0 g of *Perilla leaf* powder and dissolve it in an ethanol solution with a specific volume fraction, maintaining a defined liquid–solid ratio. Subsequently, subject the mixture to extraction for 60 s using an experimental flash evaporator, followed by ultrasound treatment at 60 °C for 10 min and centrifugation at 4500 R/min for 15 min. The supernatant was filtered and extracted twice, and the filtrate was combined.

Single-factor test: liquid to material ratio (10:1, 15:1, 20:1, 25:1, 30:1), ethanol volume fraction (40%, 50%, 60%, 70%, 80%), sonication temperature (40 °C, 50 °C, 60 °C, 70 °C, 80 °C), sonication time (0 min, 5 min, 10 min, 15 min, 20 min) and flash extraction time (20 s, 40 s, 60 s, 80 s, 100 s) were selected as single factors to investigate its effect on the extraction rate of total flavonoids from *Perilla leaves*.

On the basis of single factor, the Box-Behnken response surface method response optimization test was carried out with liquid–solid ratio, ethanol volume fraction and ultrasonic temperature as the factors. Table 3 shows the different detection factors and their corresponding levels.

#### 3.2.3. Isolation and Purification of Flavonoids from *Perilla leaves*

##### Pretreatment of Resin

The four types of macroporous resins (D101, x-5, HPD450, and nka-9) were weighed and soaked in 95% ethanol for 24 h. After the macroporous resin was fully swollen, it underwent thorough washing with distilled water until all traces of alcohol were completely eliminated. Then soaked with 5% sodium hydroxide and 5% hydrochloric acid for 2–4 h, washed with distilled water to neutral and set aside.

##### Resin Screening

Totals of 5.0 g of pretreated macroporous resins were accurately weighed and placed in a 150 mL stoppered conical flask, followed by the addition of 30 mL of the sample solution. The samples were adsorbed at 160 r/min at 25 °C for 24 h to determine the flavonoid content in the solution. Then, the resin was subsequently rinsed with distilled water until it had no alcohol taste, and 30 mL of 70% ethanol was added to analyze the flavonoid content in the analytical solution. The determination of the adsorption rate was conducted utilizing the subsequent equation:

A = (C_0_ − C_1_)/C_0_ × 100% (3)

In the context of this study, A represents the percentage of adsorption rate; C_0_ is the initial concentration value of the sample solution in mg/mL (mg/mL); and C_1_ refers to the concentration of flavonoids in the solution once adsorption equilibrium is reached.

The formula for calculating the rate of resolution is as stated below:

B = C_2_ × V_2_/[V_1_ × (C_0_ − C_1_)] × 100% (4)

B represents the percentage of resolution rate; C_2_ denotes the concentration of flavonoids in the resolution solution (mg/mL); V_1_ refers to the volume of the solution used for adsorption (mL); V_2_ indicates the volume of the resulting eluate (mL).

##### Dynamic Curve of HPD450 Resin

The pretreated HPD450 resin (5.0 g) was mixed with 30 mL 0.06 mg/mL extract, and the resin was adsorbed at 160 r/min in a 25 °C constant temperature shaker for 24 h. The flavonoid content was determined by sampling every 1 h, and the adsorption dynamic curve was drawn. The resin was rinsed with distilled water and resolved in 70% ethanol. The flavonoid content was determined by hourly sampling, and an analytical dynamic curve was subsequently constructed.

##### Optimization of Enrichment Process for HPD450 Resin

The column was loaded by wet method, and the concentration of loading solution was fixed at 0.06 mg/mL. The effects of loading volume (10 mL, 15 mL, 20 mL, 25 mL, 30 mL) and eluent concentration (50%, 60%, 70%, 80%, 90%) on the extraction rate of flavonoids in *Perilla leaves* was investigated. Additionally, the influence of the volume of resolving liquid on the mass concentration of flavonoids was examined.

#### 3.2.4. Identification of Flavonoids in *Perilla leaves*

The composition of flavonoids in *Perilla leaves* was analyzed by liquid chromatography. The chromatographic conditions were as follows: Agilent C18 column (100 mm × 4.6 mm, 1.8 μm, Agilent Company, Santa Clara, CA, USA); flow rate 1.0 m L/min, column temperature 30 °C, injection volume 10 μL, detection wavelength 280 nm; mobile phase A was 0.1% phosphoric acid; mobile phase B was acetonitrile; the gradient elution process was as follows: 0 min, 90% A, 10% B; 30 min, 65% A, 35% B; 45 min, 90% A, 10% B. The gradient elution process was as follows: 0 min, 90% A, 10% B.

#### 3.2.5. Study on Antioxidant Activity

##### DPPH Free Radical Scavenging Experiment

The DPPH free radical scavenging experiment was conducted based on the content of this literature [32], 2 mL of 10–4 mol/L DPPH radical solution was added first, then 2 mL of sample solution with mass concentrations of 0.2, 0.4, 0.6, 0.8, 1.0 mg/mL and 2 mL of VC solution (ascorbic acid) with mass concentrations of 0.2, 0.4, 0.6, 0.8, 1.0 mg/mL were added, and the absorbance values were measured at 517 nm for a duration of 30 min.

DPPH clearance rate (%) = [1 − (A_1_ − A_2_)/A_0_] × 100% (5)

Type: A_0_ represents the blank control absorbance value; A_1_ corresponds to the absorbance value of the sample solution; moreover, A_2_ indicates the absorbance value of absolute ethanol and sample solution.

##### Determination of Hydroxyl Radical Scavenging Ability

•OH clearance test: referring to the literature [33], the ferrous sulfate solution (1.8 mmol/L, 2 mL) and salicylic acid-ethanol solution (1.5 mL) were sequentially added, followed by the addition of sample solutions (0.2, 0.4, 0.6, 0.8, and 1.0 mg/mL with a volume of 1 mL each) and VC solutions(ascorbic acid) (0.2, 0.4, 0.6, 0.8, and 1.0 mg/mL with a volume of ml each). On this basis, hydrogen peroxide solution (0.03%, 0.1 mL) was added and heated in 37 °C water bath for 30 min until the reaction was complete, and the absorbance value was measured at 510 nm.

•OH clearance rate (%) = [1 − (A_1_ − A_2_)/A_0_] × 100% (6)

Type: A_0_ represents the absorbance value observed in the blank control, while A_1_ refers to the absorbance value measured for the sample, and A_2_ signifies the absorbance value of the sample without hydrogen peroxide solution.

##### ABTS+• Scavenging Effect of Free Radicals

The ABTS free radical scavenging assay was conducted in accordance with the methodology described in the literature [34]. The ABTS solution (7 mmol/L, 20 mL) and potassium persulfate solution (2.45 mmol/L) were combined in 1:1 volumes and incubated in a light-restricted environment for 12–16 h. The absorbance of the resulting mixture was adjusted to achieve a value of 0.70 ± 0.02 at a wavelength of 734 nm using absolute ethanol. Subsequently, sample solutions with mass concentrations of 0.2, 0.4, 0.6, 0.8 and 1.0 mg/mL (each with a volume of 0.4 mL) along with VC solutions (ascorbic acid) with mass concentrations of 0.2, 0.4, 0.6, 0.8 and 1.0 mg/mL (also each with a volume) were prepared and combined with ABTS working solution (4 mL). Wait for 5 min and measure the absorbance at 734 nm.

(7) The clearance rate (%) = (1 − A_1_/A_0_) × 100%

Type: A_0_ is the absorbance value of blank control, A_1_ is the absorbance value of the sample.

##### Determination of Reducing Power

According to the literature [35], take 2 mL of sample solution with a mass concentration of 0.2, 0.4, 0.6, 0.8 or 1.0 mg/mL and 2 mL of VC solution(ascorbic acid) with the same mass concentration range, add 2.5 mL of PBS buffer solution with pH = 6.6 and dissolve with 1% potassium ferricyanide, and incubate in a water bath at 50 °C for 20 min. Add 1 mL 10% trichloroacetic acid solution and mix thoroughly, centrifuge for 4500 r/min for 10 min, then take 2.5 mL supernatant, add 2.5 mL distilled water and 0.5 mL 0.1% ferric chloride solution to mix, stand for 10 min, and measure the absorbance value at 700 nm.

The reduction ability of the sample is compared by A-A_0_. The larger the A-A_0_ value, the stronger the reducing capacity of the sample. A_0_ is the absorbance value of the blank control, and A_1_ is the absorbance value measured by the sample.

## 4. Conclusions

This article studies the extraction, purification and antioxidant activity of flavonoids from *Perilla leaves*. Finally, the optimal extraction conditions of flavonoids from *Perilla leaves* by flash extraction-assisted ultrasound were determined as follows: liquid–solid ratio 20:1, ethanol volume fraction 60%, ultrasonic temperature 60 °C, ultrasonic time 10 min, flash time 60 s. This method was convenient and fast, and the yield of flavonoids was high, with good effect. Through a series of static adsorption and analytical tests, HPD450 resin was determined as the appropriate material for the separation and purification of *Perilla leaf* flavonoids, and the enrichment process of HPD450 resin was optimized: the sample concentration was 0.06 mg/mL, the sample volume was 20 mL, the eluent concentration was 70% ethanol and the elution volume was 76 mL. In the in vitro antioxidant test, the order of antioxidant capacity was as follows: VC > purified > crude extract. The findings presented in this study establish a solid theoretical foundation for the advancement of bioactive compounds derived from *Perilla leaves*, thereby expanding its potential applications.

## Figures and Tables

**Table 1:** Box-Behnken design with experimental value.

The No.	Liquid/Material Ratio (mL/g)	Ethanol Volume Fraction (%)	Ultrasonic Temperature (°C)	Flavonoids Extraction Yield (mg/g)
1	20	50	70	9.04
2	15	60	70	6.77
3	20	60	60	9.82
4	15	70	60	6.57
5	20	70	50	8.99
6	25	60	70	9.35
7	20	60	60	9.76
8	20	60	60	9.78
9	20	70	70	9.19
10	15	50	60	6.75
11	25	50	60	9.03
12	25	60	50	9.08
13	20	60	60	9.81
14	25	70	60	9.43
15	20	60	60	9.79
16	15	60	50	6.66
17	20	50	50	9.06

**Table 2:** Analysis of variance (ANOVA) for the effect of extraction conditions on flavonoids yield.

Sources of Variance	Sum of Squares	Degrees of Freedom	The Mean Square	The F Value	*p* Values	Significant
model	23.90	9	2.66	1526.18	<0.0001	**
A	12.85	1	12.85	7386.47	<0.0001	
B	0.011	1	0.011	6.47	0.0385	
C	0.039	1	0.039	22.53	0.0021	
AB	0.084	1	0.084	48.33	0.0002	
AC	6.4 × 10^−3^	1	6.4 × 10^−3^	3.68	0.0966	
BC	0.012	1	0.012	6.95	0.0336	
A2	9.17	1	9.17	5271.81	<0.0001	
B2	0.58	1	0.58	333.07	<0.0001	
C2	0.52	1	0.52	298.13	<0.0001	
residual	0.012	7	1.74 × 10^−3^			
Loss of quasi	9.9 × 10^−3^	3	3.3 × 10^−3^	5.79	0.0614	
Pure error	2.28 × 10^−3^	4	5.7 × 10^−4^			
The sum of the	23.91	16				

Note: ** indicates significant.

**Table 3:** Box-Behnken test factors and levels.

Factors	The Level of Factors		
	−1	0	1
Liquid/material ratio (mL/g)	15:1	Those days	25:1
Ethanol volume fraction (%)	50	60	70
Ultrasonic temperature (°C)	50	60	70

## Data Availability

All data, tables and figures are originals.
